# Toxicity and Outcomes of Moderately Hypofractionated Radiation for Prostate Cancer With Seminal Vesicle Involvement

**DOI:** 10.1016/j.adro.2023.101252

**Published:** 2023-04-24

**Authors:** Scarlett Acklin-Wehnert, David Carpenter, Divya Natesan, R. Warren Floyd, Laura Waters, Haijun Song, W. Robert Lee, Joseph Salama, Matthew Boyer

**Affiliations:** a Department of Radiation Oncology, Durham VA Medical Center, Durham, North Carolina; b Department of Radiation Oncology, Duke University, Durham, North Carolina; c Department of Radiation Oncology, University of North Carolina, Chapel Hill, North Carolina; d Department of Internal Medicine, Wellstar Kennestone Hospital, Marietta, Georgia

## Abstract

**Purpose:**

The aim of this study was to assess the toxicity and outcomes following treatment of prostate cancer with seminal vesicle involvement (SVI) evident on magnetic resonance imaging or clinical examination with moderately hypofractionated radiation therapy (MHRT).

**Methods and Materials:**

Forty-one patients treated with MHRT to the prostate and 1 or both seminal vesicles from 2013 to 2021 at a single institution were identified and propensity score matched to 82 patients treated during the same period with prescription dose given to the prostate alone. Dosimetry of the planning target volume, bladder, and rectum were compared. Urinary and bowel toxicity were scored by National Cancer Institute Common Terminology Criteria for Adverse Events, version 5.0. Clinical outcomes including freedom from biochemical recurrence, prostate cancer–specific survival, and overall survival were assessed.

**Results:**

Of the 41 patients identified with SVI, 26.8% had SVI by clinical examination and 95.1% had high-risk prostate cancer. Compared with the cohort without SVI, treatment plans to include SVI had a larger planning target volume (152.2 vs 109.9 cc; *P* < .001), maximum point dose (107.9% vs 105.8%; *P* < .001), and volume receiving 100% of the prescription dose (143.1 vs 95.9 cc; *P* < .001). No difference in bladder dosimetric variables between cohorts was observed, but there was an increase in the rectal maximum point dose (103.9% vs 102.8%; *P* = .030) and rectal volume receiving 100% of the prescription dose (1.8 vs 1.2 cc; *P* = .016). Despite these differences, there was no difference in the cumulative incidence of grade 2+ urinary (hazard ratio [HR], 0.73; 95% CI, 0.39-1.35; *P* = .31) or bowel (HR, 0.35; 95% CI, 0.04-3.03; *P* = .34) toxicity. Freedom from biochemical recurrence (HR, 0.47; 95% CI, 0.16-1.38; *P* = .17), prostate cancer–specific survival (HR, 0.31; 95% CI, 0.04-2.49; *P* = .31), and overall survival (HR, 0.35; 95% CI, 0.10-1.16; *P* = .09) also did not differ with or without SVI, respectively.

**Conclusions:**

Treatment of SVI to prescription dose with MHRT for localized prostate cancer does not increase bowel or urinary toxicity. Similar clinical outcomes were also observed with or without SVI.

## Introduction

Prostate cancer is the most common cancer in men, with an estimated incidence of approximately 250,000 cases in the United States in 2021 and three-fourths of patients presenting with localized disease.^1^ Seminal vesicle involvement (SVI) portends a worse prognosis in these patients whether it is identified clinically,^2^ on pretreatment magnetic resonance imaging (MRI),^3^^,^^4^ or at the time of prostatectomy.^5^, ^6^, ^7^

For patients receiving definitive radiation treatment, guidelines recommend offering moderately hypofractionated radiation therapy (MHRT) at 2.5 Gy to 3 Gy per fraction for all risk groups.^8^ This recommendation is largely based on the results of 4 randomized trials showing similar rates of biochemical control and treatment-related toxicity compared with conventionally fractionated radiation at 1.8 Gy to 2 Gy per fraction.^9^, ^10^, ^11^, ^12^ With SVI, consideration should be given to treating 1 or both seminal vesicles (SVs) to the same prescribed dose as the prostate itself. The trials of MHRT, however, largely did not address treatment of SVI or the clinical outcomes of treating the SVs to the prescription dose. A multicenter, retrospective study from France did show moderate increases in bowel and urinary toxic effects compared with contemporary studies with curative dose to the SVs; however, this study was limited to patients treated with conventional fractionation, and doses to the involved SV ranged from 46 Gy to 80 Gy.^13^ Whether treating the SVs to full dose using moderately hypofractionated regimens increases toxicity has not been shown. This study assesses the outcomes of patients treated with MHRT to 1 or both SVs and whether treating the SVs to full prescription dose increases toxicity.

## Methods and Materials

### Patients

Patients with intermediate or high-risk prostate cancer based on the D'Amico classification^14^ with SVI by clinical examination or MRI treated at Durham VA Medical Center with MHRT to either 70 Gy in 28 fractions or 60 Gy in 20 fractions from August 2013 to March 2021 were identified. Patients with intermediate to high-risk prostate cancer without evidence of SVI on clinical examination or MRI and treated over the same time frame with dose painting the SVs to either 58.8 Gy in 28 fractions or 50 Gy in 20 fractions were identified as a control group. Clinically node-positive patients or those with metastatic disease were excluded. Patients with <1 year of follow-up were also excluded. This study was approved by the institutional review board at Durham VA Medical Center.

### Treatment planning and delivery

All patients were simulated in the supine position with an alpha cradle or combi-fix for immobilization. Patients were simulated and treated with recommendations for a full bladder and empty rectum. Clinical target volumes were generated including either the prostate and, if involved, 1 or both SVs or the uninvolved SV(s) alone. Clinical target volumes were expanded by either 5 mm in all directions or by 8 mm in all directions except 5 mm posteriorly to generate planning target volumes (PTVs). The high-risk PTV including the prostate and involved SV(s) was treated to the prescribed dose, either 70 Gy or 60 Gy based on the fractionation, and the uninvolved SV PTV was limited to either 58.8 Gy or 50 Gy, respectively. For patients with invasion of only 1 SV, the contralateral SV was treated to 1 of the lower doses indicated previously based on the fractionation schedule. Per institutional preference, no patients had elective treatment of the pelvis. Treatment planning with intensity modulated radiation therapy or volumetric modulated arc therapy was employed for all patients. Patients were treated with daily image guided radiation therapy with cone beam computed tomography and/or alignment to intraprostatic fiducials. Patients were followed after treatment with clinical assessment and prostate-specific antigen (PSA) evaluation every 3 to 6 months.

### Statistics

Patients with SVI were propensity score matched 1:2 to patients without SVI on examination or imaging by a nearest neighbor method with a delta of 0.2. For patient and treatment characteristics, continuous data were compared with the Wilcoxon rank sum test and categorical data with the χ^2^ test. On treatment, early and late toxicity were defined as the worst toxicity during the course of radiation treatment within 3 months or >3 months after completing radiation, respectively. Toxicity was graded retrospectively according to the National Cancer Institute Common Terminology Criteria for Adverse Events, version 5.0. Cumulative rates of grade 2+ toxicity, freedom from biochemical recurrence (FFBR), prostate cancer–specific survival, and overall survival (OS) were estimated with the Kaplan-Meier method and compared with a log-rank test. The tests were 2-tailed with a *P* value <.5 as significant. Biochemical recurrence was defined as a rise in PSA 2 ng/ml over the posttreatment nadir per the Phoenix criteria.^15^ Statistical analyses were performed with R Studio (PBC, Boston, MA).

## Results

Forty-one patients treated to either 1 or both SVs from 2013 to 2021 were propensity score matched to 82 patients without definitive treatment of either SV. The median age of all patients was 66, with 58.5% of patients African American. Twenty-seven percent of patients in the SVI cohort had evidence of SVI by clinical examination. Overall, 95% of patients had high-risk prostate cancer, and the majority were treated to 70 Gy (78%) with concurrent androgen deprivation therapy (96%). The median follow-up for all patients was 35.7 months (Table 1).

**Table 1:** Patient characteristics of the cohorts with and without SVI

Characteristic	No SVI (n = 82)	SVI present (n = 41)	Total (n = 123)	*P* value
Age, y				.623
Median (range)	66.3 (46.1-84.2)	66.6 (36.4-80.1)	66.4 (36.4-84.2)	
Race				.585
African American	47 (57.3)	25 (61.0)	72 (58.5)	
White	33 (40.2)	16 (39.0)	49 (39.8)	
Other	2 (1.6)	0 (0)	2 (1.6)	
Clinical tumor stage				<.001
T1c	43 (52.4)	14 (34.1)	57 (46.3)	
T2a	11 (13.4)	6 (14.6)	17 (13.8)	
T2c	25 (30.5)	7 (17.1)	32 (26.0)	
T3a	3 (3.7)	1 (2.4)	4 (3.3)	
T3b	0 (0)	11 (26.8)	11 (8.9)	
T4	0 (0)	1 (2.4)	1 (0.8)	
Tx	0 (0)	1 (2.4)	1 (0.8)	
PSA, ng/ml				.869
Median (range)	17.0 (1.5–152.6)	15.7 (4.4–214.6)	16.0 (1.5–214.6)	
Gleason grade group				.899
1	6 (7.3)	2 (4.9)	8 (6.5)	
2	14 (17.1)	8 (19.5)	22 (17.9)	
3	14 (17.1)	5 (12.2)	19 (15.4)	
4	27 (32.9)	16 (39.0)	43 (35.0)	
5	21 (25.6)	10 (24.4)	31 (25.2)	
Risk group				-
High	78 (95.1)	39 (95.1)	117 (95.1)	
Intermediate	4 (4.9)	2 (4.9)	6 (4.9)	
Low	0 (0)	0 (0)	0 (0)	
Androgen deprivation therapy				.511
No	4 (4.9)	1 (2.4)	5 (4.1)	
Yes	77 (95.1)	40 (97.6)	117 (95.9)	
Months of androgen deprivation therapy				.884
Median (range)	24 (0-72)	24 (6-36)	24 (0-72)	
Radiation dose/fractions				.065
60 Gy/20	14 (17.1)	13 (31.7)	27 (22.0)	
70 Gy/28	68 (82.9)	28 (68.3)	96 (78.0)	
Follow-up, mo				.157
Median (range)	36.0 (3.0–127.3)	35.7 (0.9–79.3)	35.7 (0.9–127.3)	

*Abbreviations:* PSA = prostate-specific antigen; SVI = seminal vesicle involvement.

Values shown are number (%) unless otherwise indicated.

The high-risk PTV (152.2 vs 109.9 cc; *P* < .001) and the volume of the PTV receiving the prescription dose (143.1 vs 95.9 cc; *P* < .001) were larger with SVI than without (143.1 vs 95.9 cc; *P* < .001; Table 2). In addition, the maximum point dose as a percentage of the prescribed dose to the rectum (103.9% vs 102.8%; *P* = .03) was higher with SVI (Table 2). The volume of the rectum receiving the prescription dose was also increased in the SVI cohort (1.8 vs 1.2 cc; *P* = .016); however, the bladder maximum point dose (104.8% vs 103.8%; *P* = .052) and bladder volume receiving the prescription dose (6.3 vs 5.0 cc; *P* = .119) were not significantly different with or without SVI, respectively. There was no significant difference in percentage of either the bladder or rectum volume receiving 50 Gy or 31 Gy (Table 2).

**Table 2:** Summary of doses to the PTV, bladder, and rectum in the cohorts with and without SVI

Dosimetric Factor	No SVI (n = 82)	SVI present (n = 41)	Total (n = 123)	*P* value
PTV				
Total volume, cc	109.9 (43.4–381.7)	152.2 (81.6–268.1)	124.0 (43.4–381.7)	<.001
Dmax, %	105.8 (101.8–123.4)	107.9 (104.5–109.7)	106.5 (101.8–123.4)	<.001
V100%, cc	95.9 (40.3–319.1)	143.1 (79.0–251.7)	111.6 (40.3–319.1)	<.001
Bladder				
Dmax, %	103.8 (100.9–122.0)	104.8 (102.4–108.3)	104.2 (100.9–122.0)	.052
V100%, cc	5.0 (0.3–19.1)	6.3 (0.9–24.6)	5.4 (0.3–24.6)	.119
V50Gy, cc	33.1 (5.3–92.8)	33.5 (7.0–128.7)	33.2 (5.3–128.7)	.900
V31Gy, cc	69.9 (15.5–190.7)	75.5 (14.6–258.7)	71.8 (14.6–258.7)	.384
Rectum				
Dmax, %	102.8 (99.3–119.9)	103.9 (101.3–108.0)	103.2 (99.3–119.9)	.030
V100%, cc	1.2 (0.0–5.7)	1.8 (0.0–7.0)	1.4 (0.0–7.0)	.016
V50Gy, cc	14.2 (2.4–32.3)	16.3 (4.0–64.9)	14.9 (2.4–64.9)	.164
V31Gy, cc	41.2 (10.9–73.7)	47.1 (15.0–165.4)	43.1 (10.9–165.4)	.092

*Abbreviations:* PTV = planning target volume; SVI = seminal vesicle involvement.

**Figure 1:**
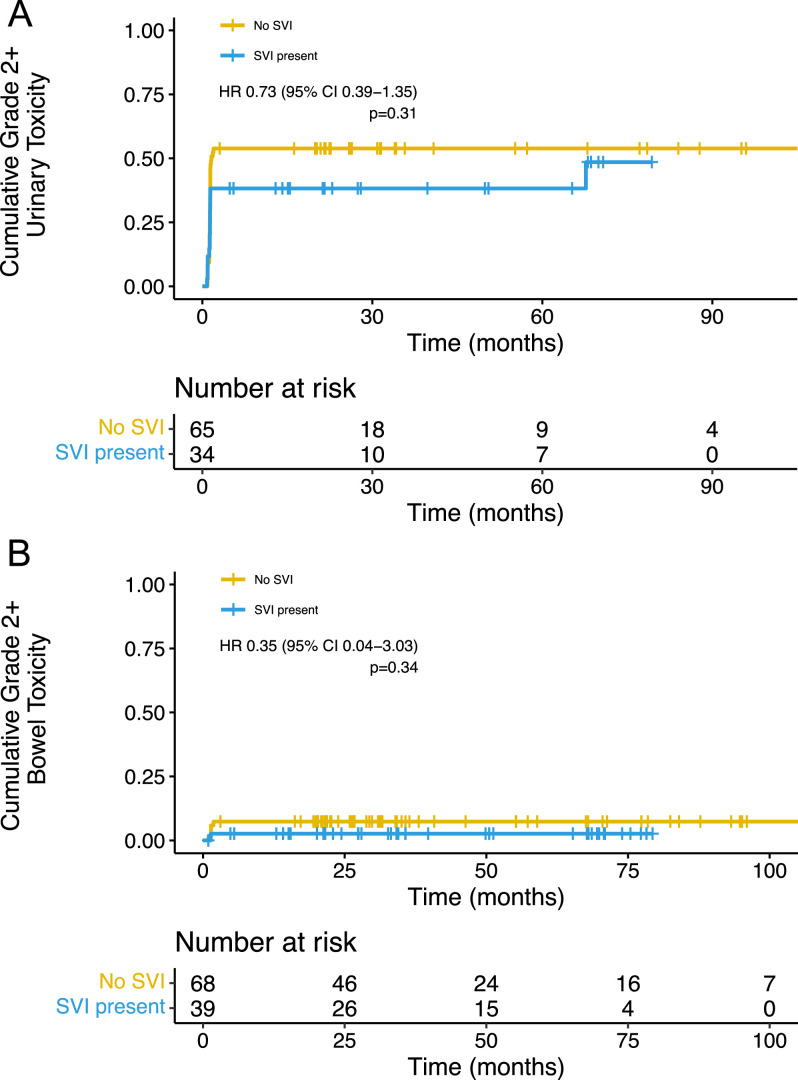
Cumulative incidence of grade 2+ (A) urinary or (B) bowel toxicity.

Given the larger treatment volume and increased maximum dose to the bladder and rectum, on treatment, acute, and late urinary and bowel toxicity were assessed. No grade 4 or 5 toxicity was observed. Late grade 3 urinary toxicity occurred in 3.5% of all patients, and no late grade 3 bowel toxicity were observed (Table E1). Cumulative rates of grade 2+ urinary (hazard ratio [HR], 0.73; 95% CI, 0.39-1.35; *P* = .31) and bowel toxicity (HR, 0.35; 95% CI, 0.04-3.03; *P* = .34) were not significantly different between patients with and without SVI (Fig. 1).

**Figure 2:**
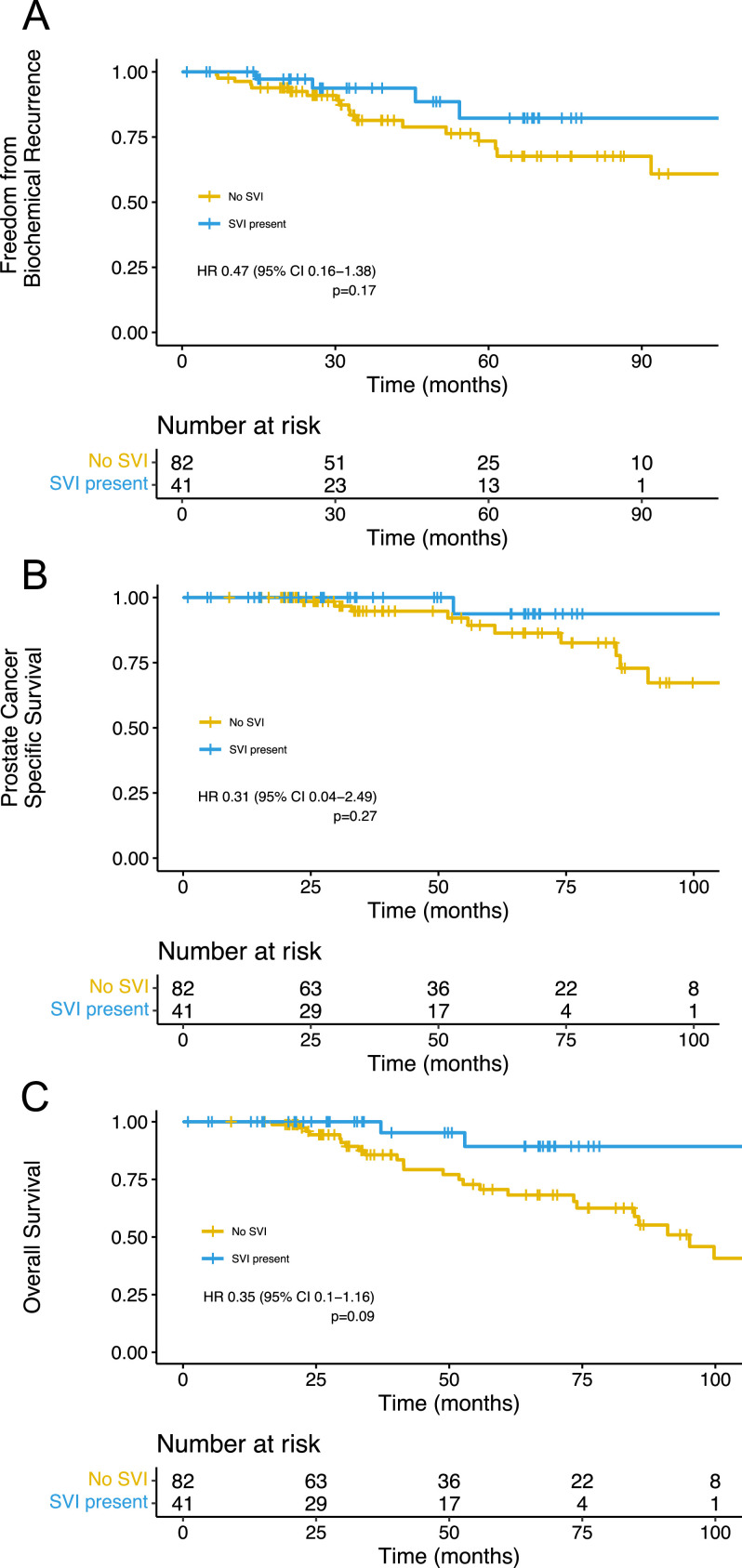
Kaplan-Meier estimates of (A) freedom from biochemical recurrence, (B) prostate cancer–specific survival, and (C) overall survival.

FFBR was similar between the cohorts with and without SVI (HR, 0.47; 95% CI, 0.16-1.38; *P* = .17; Fig. 2A). There was also no difference observed in prostate cancer–specific survival (HR, 0.31; 95% CI, 0.04-2.49; *P* = .27; Fig. 2B) or OS (HR, 0.35; 95% CI, 0.1-1.16; *P* = .09; Fig. 2C).

## Discussion

This study is the first description of toxicity rates and clinical outcomes following MHRT for definitive treatment of prostate cancer with SVI. Notably, SVI and corresponding inclusion within high-risk PTV was not associated with a significant increase in acute or late urinary or bowel toxicity. Together, these results suggest that SVI, despite correlation to larger treatment volumes and increased dose to the rectum, is not associated with higher toxicity rates following MHRT.

Overall, patients on the large phase 3 trials addressing MHRT for localized prostate cancer either did not receive treatment or received an elective dose to the SVs. The PROFIT study limited high dose to the proximal 1 cm of SVs in patients with risk of SVI >15%, Radiation Therapy Oncology Group 0415 included only favorable-risk patients and did not include the SVs in treatment volumes, and CHHiP only treated the prostate to full dose.^9^, ^10^, ^11^ In addition, all of these studies excluded patients with cT3b disease.^9^, ^10^, ^11^ In the HYPRO study, in which approximately 10% of patients had T3b disease, one-third of patients were felt to have a risk of SVI of >25% and received full dose to the SVs.^12^ The present data suggest that treatment of SVI, detected by clinical examination or by MRI, by MHRT to full prescription dose is clinically appropriate.

Expanded use of MRI in staging and treatment planning has implications on target volumes as it more effectively detects SVI. Specifically, the use of endorectal MRI was shown to increase the accuracy of detecting histologic SV invasion from 60% with digital rectal examination and 66% with transrectal ultrasound to 89%.^15^ A more recent study evaluating the staging accuracy of endorectal 3T multiparametric MRI compared with tissue histopathology illustrated similar results. In the overall cohort, sensitivity, specificity, positive predictive value, and negative predictive value for SVI detection on 3T multiparametric MRI were 75.9%, 94.7%, 62%, and 97%, respectively. Interestingly, these values further improved with radiologist expertise.^16^ In our study population, only 26.8% of patients in the SVI group were staged as T3b based on clinical examination, with the rest having evidence of SVI by MRI.

Avoidance of excessive toxicity, particularly late toxicity, is essential in clinical practice for diseases with a long natural history such as prostate cancer. Our rates of grade 2+ late urinary and bowel toxicity in the SVI cohort were 30% and 2.5%, respectively, with no acute or late grade >3 bowel toxicity. These results are similar to the outcomes reported in the PROFIT trial,^9^ where patients received 60 Gy to the prostate and proximal 1 cm of the SVs if their estimated risk of SVI was >15% based on Partin's nomogram. Defined as the worst grade from 6 months onward, their rates of late grade 2+ bowel and urinary toxicity were 22.2% and 8.9%, respectively. Moreover, our results compare favorably to Goupy et al, who reported toxicity rates of conventional radiation therapy as definitive treatment for patients with T3b prostate cancer. In that study, the rate of late grade 2+ toxicity was 31.5% and 12% for urinary and bowel toxicities, respectively.^13^

FFBR, prostate cancer–specific survival, and OS were not significantly different between the 2 cohorts in this study, likely due to a small sample size. SVI has repeatedly been shown to have a negative effect on prognosis in multiple other reports.^2^, ^3^, ^4^, ^5^, ^6^, ^7^ In addition to the small sample size of 123 patients total, the results here should be considered in the setting of limited median follow-up of 36 months, and differences in other confounding characteristics including, but not limited to, PSA, Gleason grade group, and duration and timing of androgen deprivation therapy. Similarly, the present analysis may have been underpowered to detect a clinical meaningful difference in toxicity rates, particularly with respect to the significant, but overall small, differences in rectal dosimetry. Larger retrospective studies or prospective studies including patients with SV invasion are necessary to address relatively small changes in toxicity or outcomes that may not have been captured by this report.

Additional limitations should also be considered. The data presented is that of a single-center retrospective cohort of 41 patients with SVI. Most patients in this study were staged with a bone scan and computed tomography of the abdomen/pelvis, not positron emission tomography–based imaging, which would be expected to have improved accuracy for diagnosing regional or distant metastatic disease.^16^ Patients on this study with more advanced disease were not treated with additional androgen receptor signaling inhibitors, which have been shown to improve metastasis-free survival.^17^ Finally, this study only includes patients treated with moderately hypofractionated schedules in 20 or 28 fractions and does not include patients treated with ultra hypofractionation, which has been shown to be noninferior in terms of failure-free survival for patients with intermediate to high-risk disease.^18^

## Conclusion

Prescription doses can be delivered to involved SVs with MHRT without increasing urinary or bowel toxicity compared with full dose prescribed to the prostate alone.

## Appendix Supplementary materials

T3b Supplement Image, application 1
