# Supplementary material for: Toxicity and Outcomes of Moderately Hypofractionated Radiation for Prostate Cancer With Seminal Vesicle Involvement
Source: Adv Radiat Oncol. 2023 Apr 24;8(5):101252. doi: 10.1016/j.adro.2023.101252 (PMC10318209; doi:10.1016/j.adro.2023.101252)
Supplement: T3b Supplement [file mmc1.docx]

|  | | **No SVI (n=82)** | **SVI present (n=41)** | **Total (n=123)** | **p value** |
| --- | --- | --- | --- | --- | --- |
| **GU Toxicity** | |  |  |  |  |
| *On Treatment* | |  |  |  | 0.139 |
|  | Grade 0 | 25 (30.5) | 10 (24.4) | 35 (28.5) |  |
|  | Grade 1 | 22 (26.8) | 18 (43.9) | 40 (32.5) |  |
|  | Grade 2 | 30 (36.6) | 13 (31.7) | 43 (35.0) |  |
|  | Grade 3 | 5 (6.1) | 0 (0.0) | 5 (4.1) |  |
| *Acute* | |  |  |  | 0.230 |
|  | Grade 0 | 31 (37.8) | 13 (31.7) | 44 (35.8) |  |
|  | Grade 1 | 13 (15.9) | 13 (31.7) | 26 (21.1) |  |
|  | Grade 2 | 34 (41.5) | 14 (34.1) | 48 (39.0) |  |
|  | Grade 3 | 4 (4.9) | 1 (2.4) | 5 (4.1) |  |
| *Late* | |  |  |  | 0.822 |
|  | Grade 0 | 29 (38.7) | 19 (47.5) | 48 (41.7) |  |
|  | Grade 1 | 19 (25.3) | 9 (22.5) | 28 (24.3) |  |
|  | Grade 2 | 24 (32.0) | 11 (27.5) | 35 (30.4) |  |
|  | Grade 3 | 3 (4.0) | 1 (2.4) | 4 (3.5) |  |
|  |  |  |  |  |  |
| **GI Toxicity** | |  |  |  |  |
| *On Treatment* | |  |  |  | 0.798 |
|  | Grade 0 | 68 (82.9) | 36 (87.8) | 104 (84.6) |  |
|  | Grade 1 | 9 (11.0) | 4 (9.8) | 13 (10.6) |  |
|  | Grade 2 | 4 (4.9) | 1 (2.4) | 5 (4.1) |  |
|  | Grade 3 | 1 (1.2) | 0 (0.0) | 1 (0.8) |  |
| *Acute* | |  |  |  | 0.023 |
|  | Grade 0 | 54 (67.5) | 38 (92.7) | 92 (76.0) |  |
|  | Grade 1 | 14 (17.5) | 2 (4.9) | 16 (13.2) |  |
|  | Grade 2 | 11 (13.8) | 1 (2.4) | 12 (9.9) |  |
|  | Grade 3 | 1 (1.2) | 0 (0.0) | 1 (0.8) |  |
| *Late* | |  |  |  | 0.213 |
|  | Grade 0 | 67 (88.2) | 39 (97.5) | 106 (91.4) |  |
|  | Grade 1 | 6 (7.9) | 1 (2.4) | 7 (6.0) |  |
|  | Grade 2 | 3 (3.9) | 0 (0.0) | 3 (2.6) |  |

**Supplemental Table 1**. Maximal genitourinary (GU) and gastrointestinal (GI) toxicity graded by CTCAE 5.0 criteria for patients on treatment, within 3 months of completing treatment (Acute), or at greater than 3 months from completing treatment (Late).
